# Advances in the clinical diagnosis of lung cancer using contrast-enhanced ultrasound

**DOI:** 10.3389/fmed.2025.1543033

**Published:** 2025-03-19

**Authors:** Jian-wei Huang, Hai Zeng, Quan Zhang, Xiao-yu Liu, Chong Feng

**Affiliations:** ^1^Department of Chest Surgery, The Affiliated Hongqi Hospital of Mudanjiang Medical University, Mudanjiang, China; ^2^Department of General Surgery, The Affiliated Hongqi Hospital of Mudanjiang Medical University, Mudanjiang, China; ^3^Department of Ultrasound, The Affiliated Hongqi Hospital of Mudanjiang Medical University, Mudanjiang, China

**Keywords:** lung cancer, contrast-enhanced ultrasound, diagnosis, multimodal imaging, advance

## Abstract

Lung cancer (LC) remains one of the leading causes of cancer-related mortality worldwide, emphasizing the urgent need for innovative diagnostic tools to improve early detection and patient outcomes. Contrast-enhanced ultrasound (CEUS) has emerged as a promising complement to conventional imaging modalities, offering distinct advantages such as real-time dynamic imaging, cost-effectiveness, and the absence of ionizing radiation. By enhancing the visualization of tumor vascularization, CEUS enables differentiation between benign and malignant pulmonary nodules while providing valuable insights into tumor angiogenesis, a hallmark of malignancy, and therapeutic response. Additionally, CEUS demonstrates utility in assessing regional lymph nodes, detecting distant metastases, and analyzing blood flow dynamics through quantitative methods such as time-intensity curve analysis. Despite these benefits, certain limitations persist, including reduced efficacy in imaging deep-seated lesions, variability due to patient-specific physiological factors, and dependency on operator expertise. However, advancements in targeted contrast agents, integration with multimodal imaging techniques, and the application of artificial intelligence hold significant potential to address these challenges. This review systematically evaluates the clinical applications, advantages, and limitations of CEUS in LC diagnosis, providing a comprehensive understanding of its role in modern precision oncology. Furthermore, it highlights future research directions aimed at enhancing diagnostic accuracy, improving clinical workflows, and expanding the adoption of CEUS in routine practice.

## 1 Introduction

Lung cancer (LC) ranks among the most prevalent and fatal malignancies worldwide, presenting a significant global health burden (1). According to the World Health Organization (WHO), over 2.09 million new cases of LC are diagnosed annually, and the disease is responsible for more than 1.76 million deaths each year (1). This accounts for approximately 18.4% of all cancer-related fatalities (1). The persistently high mortality rates are primarily attributed to challenges in early detection and the predominance of advanced-stage diagnoses at the time of clinical presentation. While notable advancements in screening techniques and therapeutic strategies have been made, the overall five-year survival rate for LC remains dismally low, averaging less than 20% in many countries (1). This underscores the pressing need for innovative, accurate, and non-invasive diagnostic modalities to improve early detection rates and overall prognosis.

Currently, traditional imaging techniques such as chest X-rays, computed tomography (CT), magnetic resonance imaging (MRI), and positron emission tomography (PET) are routinely employed for LC diagnosis and staging (2–6). These modalities, while invaluable for tumor localization and tissue characterization, are not without limitations. CT imaging, for instance, exposes patients to ionizing radiation, raising concerns about cumulative radiation risks, particularly in high-risk populations undergoing routine surveillance (3, 4). Similarly, MRI, despite its superior soft tissue resolution, is associated with high costs and limited accessibility due to operational complexities. PET, often combined with CT (PET-CT), provides metabolic insights by detecting increased glucose uptake in malignant tissues, which is particularly useful for staging and identifying distant metastases (7). However, PET-CT is also associated with high costs, limited accessibility, and exposure to ionizing radiation, which may limit its use in certain patient populations. Moreover, these modalities may exhibit suboptimal sensitivity and specificity in distinguishing benign from malignant lesions, often resulting in diagnostic ambiguities and unnecessary invasive procedures such as biopsies (3–6). These challenges highlight an urgent need for more advanced imaging tools that address the limitations of existing technologies and enhance diagnostic precision.

Contrast-enhanced ultrasound (CEUS) has emerged as a complementary imaging technique in this context, offering unique advantages that provide additional information beyond what is offered by X-ray, CT, MRI, and PET-CT (8). By utilizing intravenous microbubble-based contrast agents, CEUS amplifies blood flow visualization and enhances the depiction of microvascular structures, enabling real-time dynamic assessment of tumor vascularity (9). This capability is particularly relevant in evaluating tumor angiogenesis, a hallmark of malignancy, and distinguishing benign from malignant pulmonary lesions (10, 11). CEUS is further characterized by its non-invasive nature, radiation-free operation, and bedside applicability, making it a practical and patient-friendly diagnostic option (12, 13). These attributes position CEUS as a valuable tool in LC diagnosis, particularly when integrated with other imaging modalities (14).

This review aims to systematically examine the clinical research progress of CEUS in the diagnosis of LC, with an emphasis on its applications in lesion characterization, differentiation between benign and malignant pulmonary nodules, and therapeutic response monitoring. Through a detailed analysis of its technical features, diagnostic accuracy, and clinical advantages, the review seeks to elucidate how CEUS complements conventional imaging methods. Additionally, this study will highlight ongoing research challenges and emerging areas of interest, such as the integration of CEUS with other imaging modalities and its role in precision oncology. By synthesizing current evidence, this review endeavors to provide a comprehensive understanding of CEUS as a diagnostic tool and outline future directions for its application in clinical practice. Furthermore, it aims to offer actionable insights for researchers and clinicians, facilitating the development of standardized protocols and broader adoption of CEUS in modern healthcare settings.

## 2 Search strategy and study selection

### 2.1 Search strategy

A systematic search was performed to identify relevant records examining the application of CEUS in the clinical diagnosis of LC. The search strategy employed a combination of key terms, including “lung cancer,” “lung neoplasm,” “pulmonary cancer,” “contrast-enhanced ultrasound,” and “CEUS.” The searches were performed in the Web of Science and PubMed databases, covering studies published from the inception of these databases to November 2024. Only articles published in English were included to ensure consistency and broad accessibility of the reviewed material. To enhance the thoroughness of the search, reference lists of identified studies were manually screened to identify additional relevant records.

### 2.2 Inclusion criteria

Studies were deemed eligible for inclusion based on the following criteria: (1) the primary focus was on the application of CEUS in the clinical diagnosis of LC; (2) the study design included clinical studies, such as randomized controlled trials or observational studies, that directly investigated the use of CEUS in diagnosing LC; and (3) the studies were peer-reviewed and published in reputable journals in the English language.

### 2.3 Exclusion criteria

Studies were excluded if they met any of the following criteria: (1) they did not directly address the application of CEUS in the clinical diagnosis of LC; (2) they were duplicate publications; (3) they did not fall under the category of clinical trials or observational studies; or (4) conference abstracts, or unpublished studies that had not undergone peer review, to ensure the inclusion of academically rigorous and reliable research.

### 2.4 Study selection

The study selection process was carried out in three systematic phases to ensure methodological rigor and consistency. First, two independent authors screened the titles and abstracts of all retrieved studies based on the predefined inclusion and exclusion criteria. Second, studies that met the criteria during the initial screening underwent a comprehensive full-text review to verify their eligibility. Finally, any discrepancies between the authors were resolved through discussion, and if consensus could not be reached, a third author was consulted to make the final decision.

**Figure 1:**
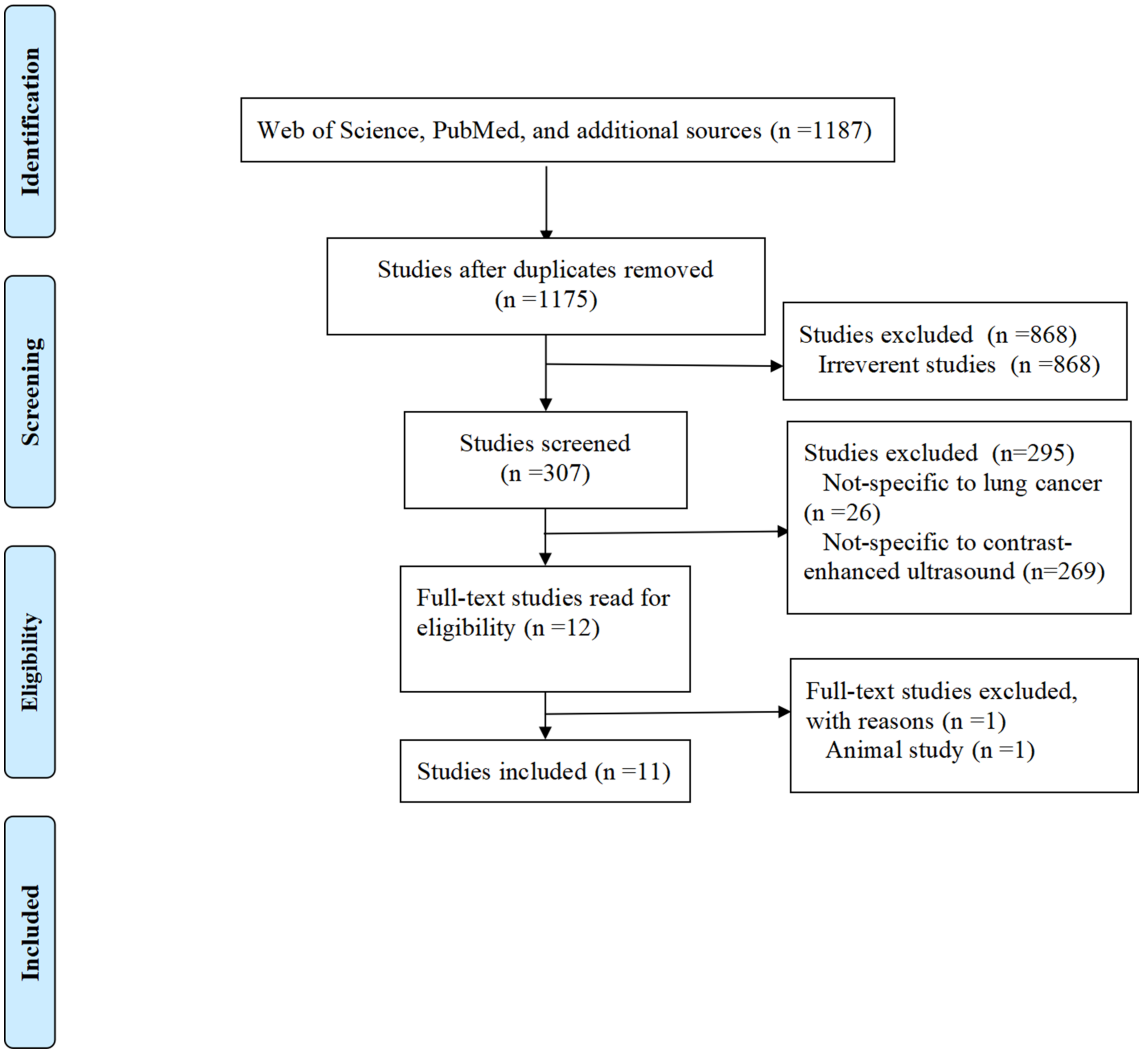
Flowchart of study selection.

A total of 1,187 records were identified through a comprehensive search. Following a rigorous screening process, 12 duplicate studies and 868 irrelevant records were excluded (Figure 1). Subsequently, 26 studies unrelated to LC and 269 studies unrelated to CEUS were further excluded. A detailed full-text review was then conducted for 12 studies that met the predefined inclusion criteria. Of these, 11 observational studies were ultimately included in the analysis, while one study was excluded because it involved animal research (Figure 1).

## 3 Application of CEUS in LC diagnosis

### 3.1 Identification and classification of LC lesions

CEUS has emerged as an essential diagnostic modality for the identification and classification of LC lesions (Table 1). By providing real-time dynamic imaging, CEUS helps distinguish malignant from benign lesions, which is critical for determining the appropriate treatment approach. A study by Wang et al. demonstrated the utility of microflow imaging in peripheral LC, where malignant lesions exhibited irregular and chaotic blood flow patterns compared to the more regular vascular structures found in benign lesions (15) (Table 1). This capability to distinguish between malignant and benign lesions is essential, as it facilitates early detection and ensures timely clinical intervention. Additionally, Sperandeo et al. (16) observed that while CEUS offers valuable insights into tumor vascularization, it is less effective in differentiating between LC and community-acquired pneumonia, underscoring the need for complementary diagnostic methods (Table 1).

**Table 1:** Summary of included studies.

Reference	Patient	Publication type	Diagnostic modality	Main findings
Wang et al. (15)	Patients with peripheral LC	Observational study	CEUS	CEUS effectively visualized neovascularization in peripheral LC, aiding in the evaluation of tumor vascularity.
Sperandeo et al. (16)	Patients suspected of community-acquired pneumonia or LC	Observational study	CEUS	CEUS showed no significant ability to differentiate between community-acquired pneumonia and LC, highlighting its limitations in certain contexts.
Li et al. (18)	Patients with primary peripheral LC	Observational study	CEUS	CEUS effectively assessed the blood supply in primary peripheral LC, providing insights into tumor vascularity and potential for targeted therapies.
Lei et al. (19)	Patients with central LC and atelectasis	Observational study	CEUS	CEUS guided needle biopsy of central LC with atelectasis, improving biopsy accuracy and visualization.
Chen et al. (20)	Patients with non-small cell LC	Observational study	CEUS	A positive correlation was found between CEUS results and microvessel density in non-small cell LC, suggesting CEUS as a useful tool for assessing tumor angiogenesis.
Wang et al. (21)	Patients with neck lymph node metastases from LC	Observational study	Conventional ultrasound combined with	Combined conventional ultrasound and CEUS quantitatively analyzed neck lymph node metastases, enhancing the diagnostic accuracy for detecting LC metastases.
Safai Zadeh et al. (22)	Patients with central LC and obstructive atelectasis	Observational study	CEUS	CEUS provided valuable diagnostic information in evaluating central LC with obstructive atelectasis, offering insights into tumor characteristics.
Yin et al. (23)	Patients with metastatic LC	Observational study	CEUS	CEUS was used to assess perfusion patterns and time-intensity curves, offering preliminary insights into evaluating metastatic lymph nodes in LC.
Li et al. (24)	Patients with non-small cell LC	Observational study	CEUS	A novel nanoparticle contrast agent improved diagnostic accuracy and visualization of LC in patients with non-small cell LC.
Schauer et al. (25)	Patients undergoing surgery for primary LC	Observational study	Io-CEUS	Io-CEUS was effective in diagnosing primary LC during surgery, aiding in real-time assessment of tumor characteristics.
Yu QD, et al. (26)	Patients with peripheral LC	Observational study	Multimodal CEUS	Multimodal CEUS helped differentiate between pathological types of peripheral LC, improving diagnostic accuracy and decision-making.

LC, lung cancer; CEUS, contrast-enhanced ultrasound; Io-CEUS, Intraoperative CEUS.

It is important to note that CEUS is particularly effective for lesions near the pleura, where ultrasound signals are less attenuated. However, its utility may be limited for deeper or non-pleural lesions due to signal interference from air-tissue interfaces and bony structures (17).

### 3.2 Tumor blood flow dynamics and microvascular characteristics analysis

CEUS provides an in-depth analysis of tumor blood flow dynamics and microvascular characteristics, key factors in assessing tumor malignancy and predicting therapeutic response (Table 1). The ability to visualize blood flow in real-time allows for the evaluation of tumor vascularization, which is directly correlated with tumor aggressiveness. Li et al. highlighted that CEUS could effectively assess blood supply in primary peripheral LC, with malignancies showing asymmetric blood flow distribution, a hallmark of aggressive tumors (18) (Table 1). Similarly, Lei et al. demonstrated that CEUS could be used to assess blood flow dynamics in central LC complicated by atelectasis, offering valuable guidance for surgical planning and treatment decisions (19) (Table 1). The detailed observation of blood flow patterns through CEUS provides critical information regarding tumor biology, supporting both diagnosis and treatment monitoring.

### 3.3 Assessment of regional lymph nodes and distant metastasis

The ability of CEUS to assess regional lymph node involvement and distant metastasis is an important application in LC staging (Table 1). In particular, CEUS provides valuable information on the vascular features of metastatic lymph nodes, offering improved diagnostic accuracy compared to conventional imaging techniques. Chen et al. demonstrated that CEUS, when combined with microvascular density analysis, could effectively identify metastatic lymph nodes, which are often challenging to detect using traditional methods (20) (Table 1). Additionally, Wang et al. (21) emphasized that the combination of conventional ultrasound and CEUS is highly effective in diagnosing metastatic cervical lymph nodes, improving diagnostic accuracy by providing a clearer view of the metastatic lesions (Table 1). This dual-modality approach enables clinicians to perform more precise staging and plan interventions accordingly.

Furthermore, CEUS plays a pivotal role in detecting distant metastases. Safai Zadeh et al. (22) investigated the use of CEUS in assessing central LC with obstructive atelectasis, showing that dynamic blood flow patterns can indicate the presence of distant metastatic lesions (Table 1). By visualizing blood flow changes, CEUS enhances the ability to detect metastasis early, thus facilitating timely therapeutic interventions.

### 3.4 Blood flow dynamics and time-intensity curve analysis

In addition to visualizing blood flow, CEUS enables a detailed quantitative analysis through time-intensity curves, providing a deeper understanding of the tumor’s microvascular environment (Table 1). Yin et al. utilized time-intensity curve analysis to assess metastatic lymph nodes from LC, finding that CEUS could provide high-resolution data that accurately reflected the perfusion characteristics of metastatic lesions, improving diagnostic accuracy in staging LC (23) (Table 1). Furthermore, Li et al. highlighted that novel nanoparticle contrast agents significantly enhance the sensitivity and specificity of CEUS, especially in detecting smaller lesions or early-stage tumors that may be missed by other imaging techniques (24) (Table 1).

### 3.5 Treatment evaluation and dynamic monitoring

Beyond diagnostic capabilities, CEUS is increasingly utilized in the evaluation of treatment efficacy and dynamic monitoring of LC therapies (Table 1). Real-time blood flow imaging enables clinicians to assess tumor response to various treatments, including chemotherapy, targeted therapy, and immunotherapy. Lei et al. found that CEUS could provide valuable insights into treatment effects, particularly in central LC, by detecting blood flow changes that correlate with tumor shrinkage and necrosis during therapy (19). Schauer et al. further expanded on this by demonstrating the value of intraoperative CEUS (Io-CEUS) in improving surgical outcomes for LC patients (25). By allowing real-time visualization of tumor margins during surgery, Io-CEUS can help surgeons detect residual disease, thereby reducing the risk of incomplete resections and improving overall patient outcomes (Table 1).

Yu et al. also explored the potential of CEUS in the context of targeted and immunotherapy, revealing that CEUS could predict treatment success by detecting changes in tumor blood flow (Table 1). These dynamic changes in blood flow can serve as early indicators of therapeutic efficacy, particularly in the early stages of treatment, when traditional methods might not show significant tumor size changes (26) (Table 1). This dynamic monitoring capability makes CEUS a powerful tool for personalized treatment planning and adjustments.

## 4 Clinical advantages and limitations of CEUS

### 4.1 Clinical advantages

CEUS has demonstrated several significant advantages in the clinical diagnosis of LC, making it a valuable complement to conventional imaging modalities.

Firstly, CEUS offers lower examination costs and real-time imaging capabilities (27). Compared to CT and MRI, CEUS is more affordable in terms of equipment maintenance and operational expenses. Additionally, CEUS does not require complex settings or facilities, making it particularly suitable for use in primary healthcare centers and resource-constrained environments (27). However, it is important to note that in some countries, CEUS may require the presence of specialized personnel, such as anesthetists, which could limit its applicability in certain primary healthcare settings. Its ability to provide real-time dynamic imaging allows for the immediate visualization of lesion blood flow and tissue characteristics, making it particularly useful for assessing tumor angiogenesis, a key feature of malignancy (28).

Secondly, CEUS reduces patient exposure to radiation (29). While CT provides high-resolution images, it involves ionizing radiation, raising concerns about cumulative exposure risks, especially for high-risk populations requiring regular imaging for monitoring (29). CEUS, in contrast, operates without ionizing radiation, offering a safer diagnostic option for patients undergoing frequent follow-up examinations.

Lastly, CEUS excels in visualizing dynamic changes in lesions. By injecting microbubble-based contrast agents, CEUS enhances the visualization of blood flow within tumors and reveals the microvascular characteristics of tissues (12). This capability is invaluable for distinguishing between benign and malignant lesions and monitoring changes in tumor blood flow and metabolism after treatment (12). In particular, CEUS has shown unique diagnostic value in assessing tumor neovascularization, tissue necrosis, and dynamic changes in lesions following chemotherapy or radiotherapy, making it an essential tool for therapeutic evaluation.

### 4.2 Limitations and challenges

Despite its numerous advantages, CEUS faces certain limitations and challenges that need to be addressed to enhance its clinical utility.

Firstly, CEUS has limited effectiveness in imaging deep-seated lesions (30). The attenuation of ultrasound signals in deep tissues reduces the quality of imaging for lesions located in the deeper regions of the lungs or in larger patients (30). Additionally, studies have reported that approximately 20–30% of pulmonary lesions may not be evaluable due to interference from air-tissue interfaces in the lungs, particularly in cases where lesions are located near the central airways or in areas with significant air content (17, 31). The bony structures of the chest wall can further hinder imaging quality. These constraints make CEUS less effective than CT or MRI for diagnosing deep or complex pulmonary lesions.

Secondly, the safety and applicability of contrast agents require further evaluation (32). While microbubble contrast agents used in CEUS are generally safe, there are contraindications for certain patient populations, such as those with severe cardiopulmonary conditions or allergies to the contrast agent components (32). Additionally, the diagnostic performance of CEUS may be affected by hemodynamic abnormalities in patients, potentially increasing diagnostic uncertainty in specific clinical scenarios.

Lastly, CEUS relies heavily on operator expertise and lacks standardized protocols (33). The accuracy and reliability of CEUS examinations depend significantly on the skill level of the operator, including selecting the appropriate probe, adjusting imaging parameters, and interpreting dynamic images (33). This dependency on operator proficiency may result in variability in diagnostic outcomes (33). Furthermore, the absence of comprehensive standardized guidelines and diagnostic criteria limits the widespread adoption of CEUS across different healthcare institutions and settings.

## 5 Current research hotspots and challenges

### 5.1 Hotspot research areas

#### 5.1.1 CEUS applications in molecular subtyping of LC

Molecular subtyping of LC has become essential in the era of precision medicine, as it directly influences treatment strategies and prognosis. CEUS shows potential in supporting molecular diagnostics by providing real-time imaging data correlated with tumor biology (34). CEUS enables detailed analysis of vascularization patterns and perfusion dynamics, which are often associated with the molecular and histological characteristics of lung tumors. For instance, differences in vascular density and perfusion parameters, such as peak intensity and time-to-peak, have been linked to molecular markers like vascular endothelial growth factor expression. Current studies are investigating the integration of CEUS imaging metrics with molecular profiles to develop non-invasive tools for tumor classification (35). Such approaches could reduce reliance on invasive biopsy procedures, facilitating early and accurate molecular diagnosis.

#### 5.1.2 Combining CEUS with ultrasound elastography for enhanced diagnosis

The combination of CEUS with ultrasound elastography represents a novel diagnostic strategy for LC. CEUS excels in visualizing tumor vascularity and perfusion, while elastography measures tissue stiffness, which is often elevated in malignant lesions (36). By integrating these two modalities, clinicians can obtain complementary data that enhances diagnostic confidence, particularly for peripheral pulmonary nodules or lesions with ambiguous features on conventional imaging (36). Preliminary evidence suggests that combining these technologies improves sensitivity and specificity in distinguishing benign from malignant lung nodules. Research is ongoing to standardize the combined use of CEUS and elastography, focusing on protocol optimization and validation in larger clinical trials. The synergy between these techniques could establish a new standard for non-invasive LC diagnostics.

### 5.2 Challenges in current research

#### 5.2.1 Diagnostic sensitivity for small pulmonary nodules

Detecting small pulmonary nodules, particularly those under 1 cm in diameter, remains a significant challenge for CEUS. The technique’s resolution limits its ability to visualize the weak blood flow and subtle vascularization features typically associated with early-stage LC (37). Small nodules often lack the well-defined neovascularization seen in larger tumors, further complicating their detection. Additionally, the acoustic shadowing and scattering caused by lung tissue and air interfaces can hinder image clarity. To address these issues, researchers are exploring advancements in probe technology, high-sensitivity contrast agents, and machine learning-based imaging analysis to enhance detection capabilities. However, these innovations require extensive validation before they can be adopted in clinical practice.

#### 5.2.2 Variability due to patient pathophysiological characteristics

Patient-specific factors, such as lung anatomy, comorbid conditions, and systemic vascular dynamics, can significantly influence the reliability of CEUS imaging (12). For example, patients with chronic obstructive pulmonary disease or emphysema exhibit altered pulmonary architecture and vascular patterns, which may lead to misinterpretation of imaging findings. Similarly, systemic diseases like heart failure or altered hemodynamics can impact the distribution and clearance of contrast agents, reducing diagnostic accuracy. Developing personalized CEUS protocols tailored to account for individual physiological variability is essential for addressing these challenges (12). Furthermore, integrating CEUS results with other diagnostic data, such as laboratory markers and clinical history, could improve overall diagnostic precision in diverse patient populations.

## 6 Future directions for CEUS in LC diagnosis

### 6.1 Development and application of novel contrast agents

The advancement of novel contrast agents is central to enhancing the diagnostic efficacy of CEUS in LC (32). Current microbubble-based agents are effective for imaging vascular structures but lack specificity for distinguishing malignant from benign lesions (32). Targeted contrast agents, designed to bind specific tumor biomarkers such as vascular endothelial growth factor or epidermal growth factor receptor, offer a promising solution. These agents can highlight tumor-specific microenvironments, improving the sensitivity and specificity of CEUS for early-stage LC detection. Furthermore, they enable molecular imaging, offering insights into tumor biology that can guide personalized treatment planning. The development of biocompatible, long-circulating, and stable agents remains a priority for clinical translation. Research should also explore the safety profiles of these agents, particularly for patients with comorbid conditions.

### 6.2 Integration of multimodal imaging

The integration of CEUS with other imaging modalities, such as CT, MRI, and positron emission tomography-CT (PET-CT), represents a significant advancement in LC diagnostics (26). Each modality provides unique strengths: CT offers detailed anatomical imaging, MRI excels in soft tissue contrast, and PET-CT delivers metabolic insights (4, 5). CEUS adds real-time, dynamic blood flow visualization without radiation exposure, complementing the static capabilities of these other modalities. Multimodal integration can enhance preoperative staging, enabling comprehensive assessments of tumor size, vascularity, and metastatic spread. Future directions include developing imaging systems that seamlessly fuse CEUS data with CT, MRI, or PET-CT images, along with standardized protocols for joint applications. These advancements could improve diagnostic accuracy while reducing the need for invasive procedures.

### 6.3 Applications of artificial intelligence and big data

Artificial intelligence (AI) and big data analytics hold transformative potential for CEUS in LC diagnostics. AI-powered algorithms can process large volumes of CEUS data to identify patterns and anomalies that may not be discernible to human observers (38). For instance, machine learning models can analyze key CEUS parameters, such as peak enhancement, time-to-peak, and wash-out dynamics, to predict malignancy (39). AI can also standardize image interpretation, reducing operator dependency and variability in diagnostic outcomes. Additionally, integrating CEUS imaging data with electronic health records and genomic information through big data platforms could facilitate population-level analyses, refining diagnostic criteria and enabling more personalized approaches. Future research should focus on training AI systems with diverse datasets to ensure their applicability across different patient populations and healthcare settings.

### 6.4 Personalization and precision medicine

Personalized and precision medicine approaches represent the future of CEUS applications in LC diagnosis. Tailoring CEUS protocols to individual patient characteristics—such as tumor size, vascularity, and location—can optimize diagnostic outcomes (40, 41). For instance, patients with peripheral nodules may benefit from high-sensitivity contrast agents, while advanced imaging techniques could address challenges associated with deep-seated lesions. Combining CEUS findings with genomic, proteomic, and metabolomic data can further enhance its precision, offering a holistic view of tumor heterogeneity (40, 41). Additionally, integrating patient-specific factors, such as comorbidities and risk profiles, into CEUS workflows can improve clinical decision-making. Collaborative efforts between radiologists, oncologists, and data scientists will be essential for translating these personalized approaches into routine clinical practice.

## 7 Summary

CEUS has made significant strides in LC diagnosis, offering high-resolution imaging and effective assessment of tumor vascularization and metastasis. Compared to traditional modalities like CT and MRI, CEUS is non-invasive, cost-effective, and convenient, making it a valuable tool for early diagnosis and staging. However, challenges remain, particularly regarding standardization and interpretation accuracy. Future research should focus on improving CEUS precision and sensitivity, as well as exploring its integration with other imaging techniques for comprehensive LC management. Additionally, the growing application of AI holds promise for enhancing CEUS’s diagnostic accuracy and efficiency, further strengthening its clinical utility.
